# The relationship between sperm viability and DNA fragmentation rates

**DOI:** 10.1186/s12958-015-0035-y

**Published:** 2015-05-14

**Authors:** Mary K Samplaski, Apostolos Dimitromanolakis, Kirk C Lo, Ethan D Grober, Brendan Mullen, Alaina Garbens, Keith A Jarvi

**Affiliations:** Division of Urology, Department of Surgery, Mount Sinai Hospital, University of Toronto, Toronto, ON Canada; Department of Pathology, Mount Sinai Hospital, University of Toronto, Toronto, ON Canada; Institute of Medical Sciences, University of Toronto, Toronto, ON Canada; Lunenfeld-Tanenbaum Research Institute, Mount Sinai Hospital, Toronto, ON Canada

**Keywords:** DNA fragmentation, Viability, Prediction, Semen

## Abstract

**Background:**

In humans, sperm DNA fragmentation rates have been correlated with sperm viability rates. Reduced sperm viability is associated with high sperm DNA fragmentation, while conversely high sperm viability is associated with low rates of sperm DNA fragmentation. Both elevated DNA fragmentation rates and poor viability are correlated with impaired male fertility, with a DNA fragmentation rate of > 30% indicating subfertility. We postulated that in some men, the sperm viability assay could predict the sperm DNA fragmentation rates. This in turn could reduce the need for sperm DNA fragmentation assay testing, simplifying the infertility investigation and saving money for infertile couples.

**Methods:**

All men having semen analyses with both viability and DNA fragmentation testing were identified via a prospectively collected database. Viability was measured by eosin-nigrosin assay. DNA fragmentation was measured using the sperm chromosome structure assay. The relationship between DNA fragmentation and viability was assessed using Pearson’s correlation coefficient.

**Results:**

From 2008-2013, 3049 semen analyses had both viability and DNA fragmentation testing. A strong inverse relationship was seen between sperm viability and DNA fragmentation rates, with r = -0.83. If viability was ≤ 50% (n = 301) then DNA fragmentation was ≥ 30% for 95% of the samples. If viability was ≥ 75% (n = 1736), then the DNA fragmentation was ≤ 30% for 95% of the patients. Sperm viability correlates strongly with DNA fragmentation rates.

**Conclusions:**

In men with high levels of sperm viability ≥ 75%, or low levels of sperm viability ≤ 30%, DFI testing may be not be routinely necessary. Given that DNA fragmentation testing is substantially more expensive than vitality testing, this may represent a valuable cost-saving measure for couples undergoing a fertility evaluation.

## Background

Both elevated DNA fragmentation and poor viability are known to be associated with male factor infertility. These two conditions are linked, as DNA fragmentation is one of the final steps before spermatozoa death [1] and conversely, DNA breaks are one of the primary instigators of sperm apoptosis [2-4]. The literature has demonstrated a clear link between DNA fragmentation and sperm viability [5], and in groups of men with high levels of DNA fragmentation, high levels of necrospermia are also seen [5].

The “Georgetown Male Factor Infertility Study” was the first study to establish DNA fragmentation fertility data in humans [6]. Data from this study were used to establish the thresholds DNA fragmentation and fertility data. For male fertility, the sperm chromatin structure assay was used to define > 30% DNA fragmentation as “significant lack of”, 15-30% DNA fragmentation as ‘reasonable’ and < 15% DNA fragmentation for ‘high’ fertility status [6]. Subsequent studies have also demonstrated that a sperm DNA fragmentation rate of > 30% is correlated with impaired fertility outcomes. A meta-analysis of four studies, with a total of 1962 men evaluating the relationship between sperm DNA fragmentation and spontaneous and intrauterine insemination pregnancies found that men with a DNA fragmentation < 30% were more likely to achieve a pregnancy or live birth (p = 0.0001) [6-8]. The second study looked at the relationship between DNA fragmentation and in vitro fertilization pregnancies, and found that couples were ~2x more likely to become pregnant if the sperm DNA fragmentation rate was < 30% [7,9]. These studies and others, support the notion that semen samples with ≥ 30% sperm DNA fragmentation by sperm chromatin structure assay have reduced fertility [10].

Necrospermia is defined as the percentage of dead sperm in a semen analysis sample > 42%, and is usually measured by assessing the membrane integrity of the cells, as determined by a dye exclusion assay [11,12]. In spite of its relatively straightforward definition, the causes and impact of necrospermia on male fertility are still relatively poorly understood. Necrospermia is commonly seen in men with spinal cord injuries [12], infections [13], chronic medical conditions [5], and after exposure to toxic substances [14]. While intuitively it makes sense that high levels of necrospermia are associated with poorer reproductive outcomes, there is a paucity of literature to support this.

One of the primary disadvantages of DNA fragmentation testing is its cost. Compared with other male fertility testing, DNA fragmentation testing is costly, as vitality testing costs approximately $2 and DNA fragmentation testing costs approximately $250, based on internal and internet quotes [15]. These costs generally are transferred to the patient and can add further financial burden to a couple looking to conceive.

We sought to determine the relationship between sperm viability and DNA fragmentation, with the hypothesis that for some patients, sperm DNA fragmentation rates could be predicted from the sperm viability rates. This may eliminate the need for sperm DNA fragmentation testing in a subset of men, representing a cost-saving measure for some couples undergoing a fertility evaluation.

## Methods

All men presenting for a fertility evaluation from 2008-2013, and having semen analyses with both viability and DNA fragmentation testing were identified. This data was reviewed in a retrospective manner. The collection of data and the analysis of the data in this database were approved by the Research Ethics Board of the Mount Sinai Hospital with reference number 05-0161-E (collection of data) and 07-0032-E (analysis of data) respectively. The date of the approval was October 18, 2005 and October 30, 2007. All participants have signed the IRB approved informed consent form.

Semen samples were collected at Mount Sinai Hospital, at least 48 hours, but not more than 7 days, after the time of last ejaculation. Semen samples were collected between 2 and 5 days from the last ejaculation. Semen samples were assessed for volume and then analyzed for sperm count, sperm concentration and motility following the 2001 WHO criteria. These criteria are based on microscopic high-power evaluation of 200 sperm for intactness of membranes of acrosome, head, neck, midpiece and tail.

Viability was assessed within 30 minutes of ejaculation. It was measured by eosin-nigrosin assay, by dissolving 1 g of eosin with 1 g of fresh sperm and then 3 g of nigrosin [16]. The percentage of viable sperm (sperm head unstained indicating living sperm) and non-viable sperm (sperm head stained indicating dead spermatozoa) was assessed by counting a minimum of 100 spermatozoa. Replicate counts of 100 sperm on each of two slides were performed. These were then repeated if >5% difference was found.

DNA fragmentation testing was performed on a frozen prepared semen sample using the sperm chromosome structure assay, as previously described [17]. Samples were treated for 30 seconds with 400 μL of a solution of 0.1% Triton X-100, 0.15 M NaCl, and 0.08 N HCl (pH 1.2). After 30 seconds, 1.2 mL of staining buffer (6 μg/mL acridine orange, 37 mM citric acid, 126 mM Na_2_HPO_4_, 1 mM disodium EDTA, and 0.15 M NaCl, pH 6.0) was admixed to the test tube, and the sample was analyzed by flow cytometry. After excitation by a 488-nm wavelength light source, acridine orange bound to double-stranded DNA fluoresced green (515 to 530 nm) and acridine orange bound to single-stranded DNA fluoresced red (630 nm or more). Three minutes after acridine orange staining, samples were analyzed in a flow cytometry activated cell sorter (Caliburflow cytometer, Becton Dickinson, San Jose, California). A minimum of 5000 cells were analyzed by a flow cytometry activated cell sorter scan interfaced with a data handler. The proportion of cells exhibiting an abnormal emission of red fluorescence, reflecting the percentage of sperm with denatured DNA, was recorded.

The relationship between DNA fragmentation and viability was assessed using Pearson’s product moment correlation coefficient. P-values and confidence intervals were obtained by standard methods, assuming normality of the data. Statistical analysis was performed in R Foundation for Statistical Computing, version 2.15.2.

## Results

A total of 2695 men underwent semen analysis testing with both DNA fragmentation and viability assays. Some men had multiple semen analyses with DNA fragmentation and viability testing. 2438 men had 1 test, 191 men had 2 tests, 37 men had 3 tests, 15 men had 4 tests, 3 men had 5 tests, 6 men had 6 tests, and 1 man had 7 tests. A total of 3049 semen samples with both DNA fragmentation and viability testing were performed.

Of the 3049 semen samples analyzed, 47 (1.5%) had sperm viability of 0-20%, 113 (3.7%) had viability of 21-40%, 450 (14.8%) had viability of 41-60%, 1920 (63%) had viability of 61-80%, and 519 (17%) had viability of 81-100%.

Higher sperm DNA fragmentation rates were associated with lower sperm viability rates (see Table 1), and samples with the lowest sperm viability 0-20% had the highest sperm DNA fragmentation rates (70.07 ± 34.39%), and samples with the highest sperm viability rates, 81-100%, were associated with the lowest sperm DNA fragmentation rates (12.40 ± 5.61%).

**Table 1:** Semen analysis DNA fragmentation rates when grouped by viability

**Sperm viability**	**DNA fragmentation rate (mean +/- standard deviation)**
0-20%	70.07 +/- 34.39%
21-40%	58.03 +/- 28.28%
41-60%	38.19 +/- 17.95%
61-80%	20.40 +/- 10.16%
81-100%	12.40 +/- 5.61%

Higher sperm DNA fragmentation rates were associated with lower sperm viability rates.

433 (16.1%) samples had a sperm viability of ≤ 58%, the lower limit of normal according to the 2010 WHO laboratory manual guidelines [18]. For these samples, the mean DNA fragmentation was 48.65 ± 25.59%. For the 2262 samples with viability > 58%, the mean DNA fragmentation was 19.57 ± 10.58% (p < 0.001).

**Figure 1:**
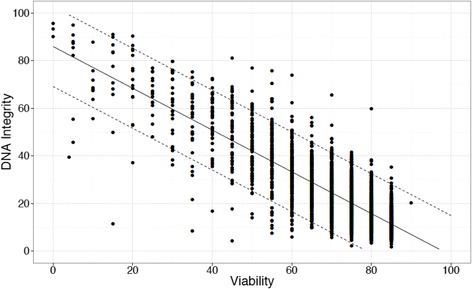
Sperm DNA fragmentation versus necrospermia. A strong inverse relationship (p < 0.001) was seen between viability and DNA fragmentation. If viability was ≥ 75%, then the DNA fragmentation was < 30% for 95% of the patients. If viability was ≤ 50% then DNA fragmentation was ≥ 30% for 95% of the samples.

A strong inverse relationship (p < 0.001) was seen between sperm viability and DNA fragmentation, with Pearson’s product-moment correlation coefficient r = -0.83 (Figure 1), t = -80.69, df = 3047, p < 0.001 and a 95% confidence interval of -0.836 to -0.814. If viability was very high (≥ 80%, n = 1104) then DNA fragmentation was consistently < 30% (100% sensitivity to predict DNA fragmentation < 30%). If viability was ≥ 75% (n = 1736), then the DNA fragmentation was < 30% for 95% of the patients (Table 2). For samples with very low viability (viability ≤ 35%, n = 91) then DFI was always ≥ 30%. If viability was ≤ 50% then DNA fragmentation was ≥ 30% for 95% of the samples (n = 310). The results of the semen analysis testing with respect to vitality and DFI are seen in Tables 3 and 4.

**Table 2:** Number of men having viability testing with DNA fragmentation ≥ or < 30% (95% confidence interval)

**Viability**	**DNA fragmentation rate (95% confidence interval)**	**Number of men (%)**
≥ 75%	< 30%	1736 (56.9%)
74-49%		1004 (32.9%)
≤ 50%	≥ 30%	310 (10.2%)

If sperm viability was ≥ 75% (n = 1736), then the DNA fragmentation was < 30% for 95% of the patients. If sperm viability was ≤ 50% then DNA fragmentation was ≥ 30% for 95% of the samples (n = 310).

**Table 3:** **Semen analysis parameters as grouped by viability**

**Viability**	**Ejaculate volume**	**Concentration**	**Motility**	**Normal morphology**	**Total motile sperm count**
≥ 75%	2.82 +/- 1.49 mL	64.23 +/- 56.05	31.89 +/- 12.62%	20.93 +/- 11.34%	58.15 +/- 61.38
74-49%	3.00 +/- 1.72 mL	54.55 +/- 58.81	22.88 +/- 11.45%	16.27 +/- 10.89%	37.44 +/- 51.82
≤ 50%	2.55 +/- 1.79 mL	38.74 +/- 44.57	8.23 +/- 7.12%	12.04 +/- 9.34%	10.31 +/- 17.88

**Table 4:** **Semen analysis parameters as grouped by DNA fragmentation**

**DNA fragmentation**	**Ejaculate volume**	**Concentration**	**Motility**	**Normal morphology**	**Total motile sperm count**
< 30%	2.84 +/- 1.53 mL	62.06 +/- 56.23	30.36 +/- 12.74%	20.06 +/- 11.31%	56.67 +/- 10.96
≥ 30%	2.93 +/- 1.84 mL	49.38 +/- 57.05	16.5 +/- 11.3%	14.2 +/- 10.71%	26.21 +/- 43.44

## Discussion

Both elevated sperm DNA fragmentation and poor sperm viability are linked to male infertility. While it is unclear in some cases which process comes first, the two are linked and the literature has demonstrated a clear link between DNA fragmentation and sperm viability [5]. Both apoptosis and necrosis result in DNA fragmentation, either by an active mechanism through apoptotic endonuclease activation or passively as in necrosis [19,20]. DNA fragmentation may also be seen in mature, viable sperm, although the mechanisms that trigger this degradation have not been fully elucidated [21,19]. After ejaculation, the incidence of sperm DNA fragmentation increases, both with duration since ejaculation [19,22] and with temperatures of 37°C or greater [23,24], but there is great inter-individual variability in these increases [19].

While reported correlations between DNA fragmentation and sperm concentration in subfertile men have varied [25-27], the literature has demonstrated a clear link between sperm DNA fragmentation and sperm viability [5]. For groups of men with high levels of sperm DNA fragmentation, high levels of necrospermia are also seen [5]. In addition, increases in DNA fragmentation rates following incubation of human spermatozoa have been shown to correlate strongly with sperm viability loss [28].

Elevated sperm DNA fragmentation rates have been positively correlated with impaired fertility, including longer times to natural conception [8], impaired embryo cleavage [29], impaired implantation rates [30], higher miscarriage rates [29], and increased risk of pregnancy loss after both in vitro fertilization and intracytoplasmic sperm injection [31]. Using the sperm chromosome structure assay, couples with a sperm DNA fragmentation of < 40% have been shown to have an odds ratio of 10× greater probability of pregnancy via natural intercourse than those with DNA fragmentation > 40% [8]. Likewise, intrauterine insemination patients have been shown to be 8.7× more likely to have a live birth if the DNA fragmentation is ≤ 27% [7]. Finally, a DNA fragmentation rate of ≥ 30% has been associated with increased spontaneous abortion rates [32]. In addition to these individual studies, two large meta-analyses using the sperm chromatin structure assay have demonstrated the clear relationship between DNA fragmentation and pregnancy. The first of these was a meta-analysis of four studies, with a total of 1962 men, found that men with a DNA fragmentation < 30% were more likely to achieve a pregnancy or live birth either spontaneously or via intrauterine insemination (p = 0.0001) [6-8]. The second found that couples were ~2× more likely to become pregnant via in vitro fertilization if their DNA fragmentation was < 30% [7,9]. Because of studies like this, sperm DNA fragmentation testing has been used increasingly as an adjunct to the standard sperm parameters [33-35].

Interestingly, while a DNA fragmentation cutoff of 30% is commonly used, there are limited studies looking at DNA fragmentation rates > 30%. We identified a single study which compared reproductive outcomes in men with sperm having DNA fragmentation rates of ≤15% compared with > 50% [33]. They found that using sperm selected by movement and morphology characteristic for intracytoplasmic sperm injection, couples with high sperm DNA fragmentation rates had similar fertilization and clinical pregnancy rates compared with sperm with low DNA fragmentation rates [33]. There are otherwise no studies looking at sperm DNA fragmentation rates > 30%, but the literature clearly demonstrates that a DNA fragmentation of > 30% negatively correlates with male reproductive outcomes.

Necrospermia is defined as a high percentage of dead sperm, as determined in our study by dye exclusion [11,12]. Living sperm have an intact cytoplasmic membrane, which is the basis for viability assays such as or dye exclusion testing, which tests sperms’ ability to resist the absorption of certain dyes, including eosin, nigrosin, or trypan blue [16]. According to the 2010 WHO laboratory manual for the examination and processing of human semen, the lower reference limit for viability (membrane-intact spermatozoa) is 58% (5th centile, 95% CI 55–63) [18].

The incidence of necrospermia in the fertile and infertile populations is poorly defined. A 2003 study of 4108 infertile men identified a prevalence of 0.7% [36], and a 2004 study estimated the prevalence of epididymal necrospermia at 0.5% in healthy men undergoing infertility work-up [37]. Both of these studies used a viability of 40% dead sperm as their cutoff definition of necrospermia, whereas we used the WHO 2010 guidelines which state that 42% dead sperm is the cutoff for necrospermia. Using a cutoff of 42%, we found an incidence of necrospermia of 16.1% in our population of infertile males, relatively high compared with the prior reported rates. For these samples, the mean DNA fragmentation was 48.65 ± 25.59%. For the 2262 samples with viability > 58%, the mean DNA fragmentation was 19.57 ± 10.58% (p < 0.001). Thus our data clearly support the notion that men with WHO defined necrospermia have higher rates of DNA fragmentation. However, in spite of its relatively straightforward definition and diagnosis, the impact and etiology of necrospermia in men with infertility is still relatively poorly understood.

This is the first study to use vitality as a predictor for the level of sperm DNA fragmentation. Given our findings, in men with sperm vitality of ≥ 75%, routine DNA fragmentation testing is unlikely to provide any additional information, as in these men sperm DNA fragmentation is very likely to be low. Likewise, for men with sperm vitality of ≤ 50% routine DNA fragmentation testing may not be required as > 95% will have sperm DNA integrity of > 30%. Based on these estimates, only 32.9% of men in our series undergoing both viability and DNA fragmentation testing would have gained additional information from DNA fragmentation testing (Table 2). In the majority of men, viability testing may predict sperm DNA fragmentation rates, allowing the couple to avoid the sperm DNA fragmentation assay and may represent a valuable cost-saving measure. While a formal cost-analysis was not performed, this may represent a valuable cost-savings measure for couples undergoing fertility evaluation, as viability testing costs approximately $2 and DNA fragmentation testing costs approximately $250, based on internal and internet quotes [15].

## Conclusions

Sperm viability correlates strongly with DNA fragmentation rates and is predictive of sperm DNA fragmentation rates. In men with high levels of sperm viability ≥ 75%, or low levels of sperm viability ≤ 30%, DFI testing may not provide additional information. Given that DNA fragmentation testing is approximately 100× more expensive than viability testing ($250 versus $2), this may represent a valuable cost-saving measure for couples undergoing a fertility evaluation.
